# Stem Cells Secretome from Oral Tissue Could Represent a Promising Therapeutic Approach in COVID-19-Disease?

**DOI:** 10.3390/ijms21186833

**Published:** 2020-09-17

**Authors:** Francesca Diomede, Guya D. Marconi, Luigia Fonticoli, Jacopo Pizzicannella, Oriana Trubiani

**Affiliations:** 1 Department of Medical, Oral and Biotechnological Sciences, University “G. d’Annunzio” Chieti-Pescara, 66100 Chieti, Italy; francesca.diomede@unich.it (F.D.); guya.marconi@unich.it (G.D.M.); luigia.fonticoli@unich.it (L.F.); 2 ASL02 Lanciano-Vasto-Chieti, “Ss. Annunziata” Hospital, 66100 Chieti, Italy; jacopo.pizzicannella@unich.it

**Keywords:** mesenchymal stem cells, human Oral Tissue Stem Cells, Coronavirus, COVID-19, immunomodulation

## Abstract

At present, severe acute respiratory syndrome coronavirus-2 (SARS-CoV-2) infection has quickly become a health emergency because no specifics vaccines or drugs, at this moment, are available. Recent studies have shown that the transplantation of mesenchymal stem cells (MSCs) into Coronavirus Disease 2019 (COVID-19) patients could represent a promising strategy for the development of new therapeutic methods. We speculate and suggest that the secretome of human Oral Tissue Stem Cells (hOTSCs), for their immunomodulatory and anti-inflammatory specific properties, could exert beneficial effects on the COVID-19 patients through an innovative aerosolisation technique. This non-invasive technique can offer multiple advantages in prophylaxis, as well as the prevention and treatment of severe epidemic respiratory syndrome with minimum risk and optimal therapeutic effects. This has the potential to create a novel pathway towards immunomodulatory therapy for the treatment of COVID-19 positive patients.

## 1. COVID-19

In mid-December 2019, a disease caused by severe acute respiratory syndrome coronavirus-2 (SARS-CoV-2) infection, which began in Wuhan, China, has spread throughout the country and currently has become a Public Health Emergency of International Concern in the other countries of the world. This pathogenic virus has been recognized in swabs executed on the throat and nose of patients who suffer from or are suspected of the disease.

At present, no vaccines or drugs have been successful for the prevention or treatment of Coronavirus Disease-19 (COVID-19) patients. Developing effective and precise approaches in preventing and curing COVID-19 is crucial, given the hugely infected population, wide transmissibility and elevated mortality rate [1].

Based on the literature, a variety of antiviral drugs, with the majority used for the treatment of HIV, hepatitis and flu symptoms, have been presently repurposed to face the novel health emergency and administered in patients with COVID-19. These drugs include lopinavir/ritonavir, remdesivir, favipiravir and tocilizumab. Advance clinical trials appear to sustain their positive effects in ameliorating patients’ clinical conditions [2]. The treatment for COVID-19 is principally related to the patients’ own immune system. Clinically, the immune responses, enhanced by SARS-CoV-2 infection, are in two distinct phases. During the first phase, a specific adaptive immune response is required to eliminate the virus and to preclude disease progression to severe stages. At this point, the strategies to enhance immune responses are definitely indispensable because the damaged cells promote innate inflammation in the lungs mediated by pro-inflammatory macrophages and granulocytes. In the second phase, lung inflammation is the primary reason for life-threatening respiratory syndromes [3].

An over-activated immune system can induce the production of a large number of inflammatory factors, in an effort to kill the virus, resulting in severe cytokine overproduction. The cytokines could stimulate organ injury, followed by edema, dysfunction of air exchange, acute respiratory distress syndrome (ARDS), acute cardiac damage, and secondary infection, which may result in death. In particular, when COVID-19 infects the upper and lower respiratory tract, it can generate mild or highly acute respiratory syndrome with a consequential release of pro-inflammatory cytokines, containing Interleukin (IL)-1β and IL-6.

The coronaviruses entry in the host cell relies on binding of the viral spike (S) proteins to cellular receptors, and on S protein priming by host cell proteases. Markus Hoffmann et al. demonstrated that SARS-CoV-2 utilizes the SARS-CoV receptor angiotensin-converting enzyme 2 (ACE2) for entry and the serine protease TMPRSS2 for S protein priming [4]. The binding of COVID-19 to the Toll Like Receptor (TLR) triggers the release of pro-IL-1β, which is cleaved by caspase-1, followed by inflammosome activation and production of active mature IL-1β, which is a mediator of lung inflammation, fever and fibrosis [3]. Suppression of pro-inflammatory IL-1 family members and IL-6 has been displayed to have a therapeutic outcome in several inflammatory illnesses, including viral infections. Moreover, cytokine IL-37 has the capability to suppress innate and acquired immune response, and additionally, can inhibit inflammation by operating on IL-18Rα receptor [5,6]. Although, in many hospitals, therapies that are normally used for the treatment of different pathologies, such as rheumatoid arthritis, AIDS, SARS, are being applied to, and test for, coronavirus pathology. To date, no certain therapy has been identified. Recently, a novel approach, involving intravenous transplantation of Mesenchymal Stem Cells (MSCs) in patients, has been reported as a promising strategy for the development a new the therapeutic methods [7,8].

Based on this knowledge, and considering the data reported on the immunomodulatory activity of stem cells, could provide a crucial strategy for the cure of COVID-19 infected patients.

## 2. Secretome from Human Oral Mesenchymal Stem Cells

In the oral cavity, important points of contact with the external environment and gateway to the respiratory and digestive systems, have been isolated six different types of hOTSCs: Exfoliated Deciduous Teeth (SHED), Stem Cells From Apical Papilla (SCAP), Periodontal Ligament Stem Cells (PDLSCs), Dental Follicle Progenitor Cells (DFPCs), Gingival Mesenchymal Stem Cells (GMSCs) and Dental Pulp Stem Cells (DPSCs) [9]. Human MSCs isolated from oral tissues, hOTSCs, possess high and for long term proliferation capability and the multipotency features that are exploited for clinical purposes, including tissue regeneration and immunomodulation [10,11]. Human MSCs, and also hOTSCs, express cell surface markers as cluster of differentiation (CD) 29, CD44, CD73, CD90, CD105 and lack the expression of CD14, CD34, CD45 and HLA (human leucocyte antigen)-DR [12,13]. In recent years, a significance significant impact on the potential use of the MSC-derived secretome, in the treatment of different diseases, has established [14,15]. In particular, the secretome from hOTSCs represents a possible candidate for a novel cell-free therapy, by overcoming the limitations and risks of cell-based therapies, including immune incompetency, carcinogenicity, condition for ex vivo cell expansion, and cost [16,17].

Hypoxic pre-conditioning has been proven to stimulate the ability of the secretome to release a multiplicity of chemokines, cytokines, and growth factors, hence, it could exhibit immunomodulatory and anti-inflammatory impacts [18].

For example, in our previous work, we demonstrated that treatment with conditioned medium (CM) from human periodontal ligament stem cells (hPDLSCs), under hypoxia (H-hPDLSCs-CM), strongly suppress experimental autoimmune encephalomyelitis (EAE), and the clinical impact decreasing principally the inflammatory pathway and the ability to react to it [19].

Moreover, the latest in vitro and in vivo studies on hOTSCs produced, not only a large quantity of cytokines, but also extracellular vesicles (EVs) with high content of anti-inflammatory mediators, which were important for the therapeutic strategy in several diseases [20,21], other than the regenerative capacity in the damaged tissues [22,23].

**Figure 1:**
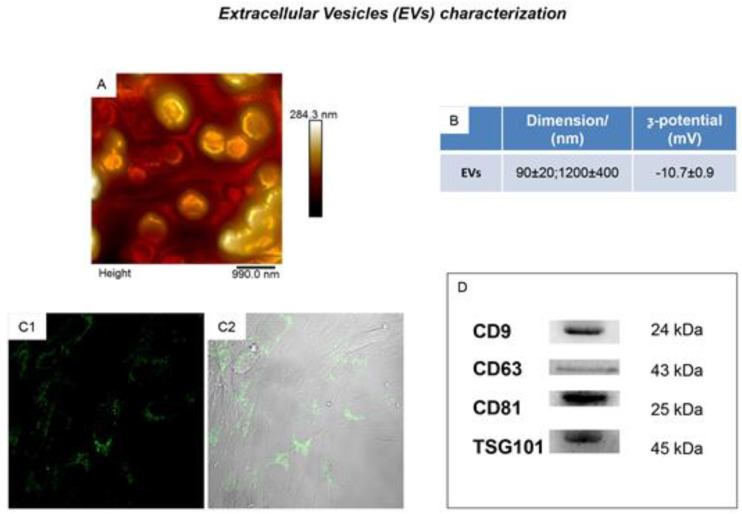
Extracellular Vesicles (EVs) characterization. (**A**) EVs morphological analysis performed by means atomic force microscope. (**B**) Average size and _ζ-potential of EVs. (**C1**,**C2**) Confocal laser scanning microscopy observations of fluorescent stained EVs cultured with hOTSCs. (**D**) Western blot analysis showed the positivity for CD9, CD63, CD81 and TSG101. Figure published in reference [25].

Extracellular vesicles (EVs) are lipid bilayer-bound vesicles secreted by cells with the characteristic to be implicated in intercellular communication [13]. The EVs are small membrane vesicles enclosing many proteins, lipids, a pool of soluble cytokines and nucleic acids such as mRNA and microRNA. EVs are characterized by the presence of specific membrane associated proteins, such as CD9, CD63, CD81, and tumor suppressor gene 101 (TSG101), as previously demonstrated [24,25] (Figure 1).

**Figure 2:**
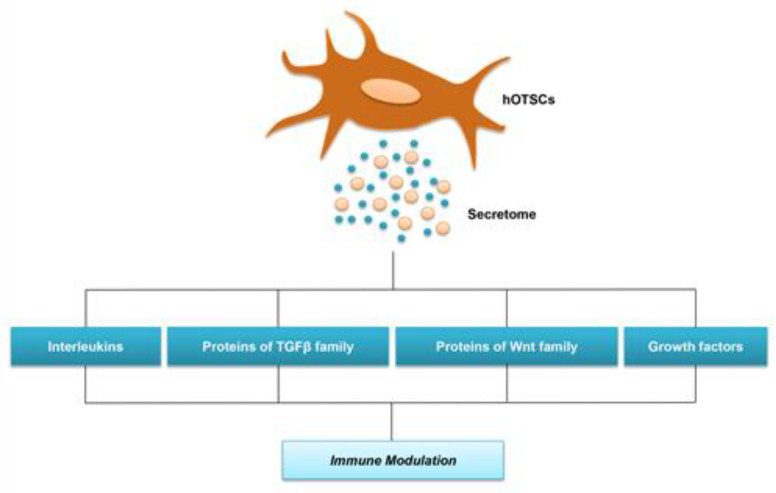
Human OTSCs secretome. Family subgroup of proteins released from the hOTSCs secretome.

EVs represent intercellular communication systems capable to interact with target cells by binding to the cell surface receptors, transferring membrane proteins, merging their membrane contents into cell recipient cell plasma membrane [20] (Figure 2 and Table 1).

Therefore, the application of hPDLSCs-derived EVs could result as a novel potential strategy for tissue engineering and regenerative medicine.

Furthermore, human periodontal ligament stem cells, spontaneously produced high levels of IL-7 and Stromal Cell-Derived factor (SDF)-1α, and are able to support the development and long-term maintenance of Bone Marrow (BM) precursor cells more efficiently than murine stromal cells and similarly to normal BM human stromal cells [26].

Cianci E. et al., 2016 [27], reported that hPDLSCs sustain polymorphonuclear neutrophil (PMN) survival and bactericidal activity. Cell-cell contact was not strictly necessary for these effects to take place, indicating paracrine immunoregulatory functions of hPDLSCs. Indeed, they release cytokines and growth factors (i.e., IL-8, VEGF) that promote PMN recruitment and survival and IL-10, which limits the pro-inflammatory activities of PMNs and improve wound repair during periodontitis. This is consistent with a proresolutive, antimicrobial profile of the hPDLSCs secretome and prompted us to evaluate the capability of these cells to produce specialized proresolving lipid mediators (SPMs), pivotal regulators of inflammation resolution. Using an established lipid mediator metabololipidomics approach, it was obtained the first evidence that hPDLSCs produce and release a broad spectrum of proresolutive mediators, including resolvin D1, D2, D5, and D6; protectin D1; maresins; and lipoxin B4; as well as prostaglandins D2, E2, and F2α. These mediators carry immunoregulatory/proresolutive bioactions by regulating granulocyte trafficking, stimulating efferocytosis and microbial clearance. Hence, the timely release of lipid mediators of inflammation and resolution may be regarded as a crucial mechanism underlying these functions. The hPDLSCs also produce large amounts of prostaglandin E2, which is a key mediator for the anti-inflammatory activity [27]. In vivo study reported that the use of hPDLSCs secretome in a murine model of experimental autoimmune encephalomyelitis (EAE), induced an anti-inflammatory and immunosuppressive effects in spinal cord and spleen other than a reverse disease progression by restoring tissue integrity via remyelinization releasing pro-inflammatory cytokines IL-17, Interferon-γ, IL-1β, IL-6 and Tumor Necrosis Factor-α. At the same time the release of anti-inflammatory IL-10 in the secretome of hPDLSCs maintained under hypoxic conditions could ameliorate EAE progression in an IL-37–dependent mechanism [19].

## 3. The Potential Use of hOMSCs-Derived Secretome in COVID-19

Starting from these assumptions, stem-cell free therapy and in particular the released secretome has been emerging as another even more interesting goal, potentially safer and cost-effective alternative for a wide range of diseases [28]. Especially secretome collected from hOTSCs, may provide a novel cell-free therapeutic approach for several diseases in order to avoid the limitations related to stem cell based therapies, which include immune incompetency, requirement for ex vivo cell expansion with important costs. Newly, it has been reported that the aerosolisation technique MicroSprayer^®^ AerosolizerModel IA-1B has been utilized for delivering airway epithelial cells in the acute lung injury. The use of MicroSprayer^®^ Aerosolizer IA-1B device has demonstrated the effectiveness of intratracheal aerosol-based MSCs delivery directly into the airway inducing an acute inflammation suppression in vivo and in vitro [29,30]. Then a novel aerosolization method, simply accessible and with low cost from oral stem cells secretome, acting directly on the oral cavity can be performed representing a first protective barrier to external agents. We consider and insinuate that the secretome of hOTSCs for their immunomodulatory and anti-inflammatory specific properties can exert therapeutic effects on the COVID-19 patients.

This non-invasive technique can offer multiple advantages in prophylaxis, prevention and treatment of COVID-19 with minimum risk and optimal therapeutic effects opening a novel pathway towards as immunomodulatory therapy for COVID-19 positive patients.

## 4. Conclusions

To conclude, stem-cell free therapy and especially the released secretome derived from hOTSCs may be considered a different therapeutic strategy, possibly harmless and cost-effective alternative for a variety of illnesses. Furthermore, we speculate that the secretome of hOTSCs for their immunomodulatory and anti-inflammatory individual belongings can exhibit beneficial outcomes on the COVID-19 patients, underlining multiple advantages in prophylaxis and prevention, opening a novel frontier for the basis for the development of an innovative approach for the co-treatment and prophylaxis of SARS-CoV-2 infection.

## Figures and Tables

**Table 1:** Family subgroup of proteins released from the hOTSCs secretome.

Secretome/EVs Content			
Interleukins (IL)	Protein of the TGF-β Family	Protein of the Wnt Family	Growth Factors
IL1B	TGFb1 Transforming Growth Factor β 1	WNT2B Wnt Family Member 2B	FGF1 Fibroblast Growth Factor 1
IL5	TGFb2 Transforming Growth Factor β 2	WNT3 Wnt Family Member 3	FGF4 Fibroblast Growth Factor 4
IL6	TGFb3 Transforming Growth Factor β 3	WNT4 Wnt Family Member 4	FGF6 Fibroblast Growth Factor 6
IL7	BMP1 Bone Morphogenetic Protein1	WNT5A Wnt Family Member 5A	FGF7 Fibroblast Growth Factor 7
IL12A	BMP2 Bone Morphogenetic Protein 2	WNT5B Wnt Family Member 5B	FGF9 Fibroblast Growth Factor 9
IL12B	BMP3 Bone Morphogenetic Protein 3	WNT7A Wnt Family Member 7A	FGF11 Fibroblast Growth Factor 11
IL15	BMP4 Bone Morphogenetic Protein 4	WNT8A Wnt Family Member 8A	FGF12 Fibroblast Growth Factor 12
IL16	BMP5 Bone Morphogenetic Protein 5	WNT9A Wnt Family Member 9A	FGF14 Fibroblast Growth Factor 14
IL17A	BMP6 Bone Morphogenetic Protein 6	WNT10A Wnt Family Member 10A	FGF18 Fibroblast Growth Factor 18
IL19	BMP7 Bone Morphogenetic Protein 7	WNT11 Wnt Family Member 11	FGF20 Fibroblast Growth Factor 20
IL21	BMP8A Bone Morphogenetic Protein 8a	WNT16 Wnt Family Member 16	FGF23 Fibroblast Growth Factor 23
IL24	BMP8B Bone Morphogenetic Protein 8b		PSPN Persephin
IL25	BMP10 Bone Morphogenetic Protein		GDNF Glial Cell Derived Neurotrophic Factor
IL27	BMP15 Bone Morphogenetic Protein 15		VEGFA Vascular Endothelial Growth Factor A
IL32	AMH Anti-Mullerian Hormone		VEGFB Vascular Endothelial Growth Factor B
IL33	GDF1 Growth Differentiation Factor 1		VEGFC Vascular Endothelial Growth Factor C
IL36B	GDF2 Growth Differentiation Factor 2		NGF Nerve Growth Factor

## Abbreviations

hOMSCs	human Oral Mesenchymal Stem Cells
SARS-CoV-2	Severe Acute Respiratory Syndrome Coronavirus-2
COVID-19	Coronavirus Disease 2019
ARDS	Acute Respiratory Distress Syndrome
IL	Interleukin
TLR	Toll Like Receptor
MSCs	Mesenchymal Stem Cells
SHED	Exfoliated Deciduous Teeth
SCAP	Stem Cells From Apical Papilla
PDLSCs	Periodontal Ligament Stem Cells
DFPCs	Dental Follicle Progenitor Cells
GMSCs	Gingival Mesenchymal Stem Cells
DPSCs	Dental Pulp Stem Cells
CD	Cluster of Differentiation
HLA	Human Leucocyte Antigen
EVs	Extracellular Vesicles
SDF-1α	Stromal Cell-Derived factor-1α
BM	Bone Marrow
PMN	Polymorphonuclear Neutrophil
VEGF	Vascular Endothelial Growth factor
SPMs	Specialized Proresolving Lipid Mediators
EAE	Experimental Autoimmune Encephalomyelitis
H	Hypoxia
CM	Conditioned Medium
